# Pre-Pubertal Children Born Post-Term Have Reduced Insulin Sensitivity and Other Markers of the Metabolic Syndrome

**DOI:** 10.1371/journal.pone.0067966

**Published:** 2013-07-01

**Authors:** Ahila Ayyavoo, José G. B. Derraik, Paul L. Hofman, Sarah Mathai, Janene Biggs, Peter Stone, Lynn Sadler, Wayne S. Cutfield

**Affiliations:** 1 Liggins Institute, University of Auckland, Auckland, New Zealand; 2 Gravida: National Centre for Growth and Development, Auckland, New Zealand; 3 Department of Obstetrics and Gynaecology, Faculty of Medical and Health Sciences, University of Auckland, Auckland, New Zealand; 4 National Women’s Health, Auckland District Health Board, Auckland, New Zealand; Virgen Macarena University Hospital, School of Medicine, Spain

## Abstract

**Background:**

There are no data on the metabolic consequences of post-term birth (≥42 weeks gestation). We hypothesized that post-term birth would adversely affect insulin sensitivity, as well as other metabolic parameters and body composition in childhood.

**Methods:**

77 healthy pre-pubertal children, born appropriate-for-gestational-age were studied in Auckland, New Zealand: 36 born post-term (18 boys) and 41 (27 boys) born at term (38–40 weeks gestation). Primary outcome was insulin sensitivity measured using intravenous glucose tolerance tests and Bergman’s minimal model. Other assessments included fasting hormone concentrations and lipid profiles, body composition from whole-body dual-energy X-ray absorptiometry, 24-hour ambulatory blood pressure monitoring, and inflammatory markers.

**Results:**

Insulin sensitivity was 34% lower in post-term than in term children (7.7 vs. 11.6 x10^-4^·min^-1^·(mU/l); p<0.0001). There was a compensatory increase in acute insulin response among post-term children (418 vs 304 mU/l; p=0.037), who also displayed lower glucose effectiveness than those born at term (2.25 vs 3.11 x10^-2^·min^-1^; p=0.047). Post-term children not only had more body fat (p=0.014) and less fat-free mass (p=0.014), but also had increased central adiposity with more truncal fat (p=0.017) and greater android to gynoid fat ratio (p=0.007) compared to term controls. Further, post-term children displayed other markers of the metabolic syndrome: lower normal nocturnal systolic blood pressure dipping (p=0.027), lower adiponectin concentrations (p=0.005), as well as higher leptin (p=0.008) and uric acid (p=0.033) concentrations. Post-term boys (but not girls) also displayed a less favourable lipid profile, with higher total cholesterol (p=0.018) and LDL-C (p=0.006) concentrations, and total cholesterol to HDL-C ratio (p=0.048).

**Conclusions:**

Post-term children have reduced insulin sensitivity and display a number of early markers of the metabolic syndrome. These findings could have important implications for the management of prolonged pregnancies. Future studies need to examine potential impacts later in life, as well as possible underlying mechanisms.

## Introduction

Post-term birth (≥42 weeks gestation) remains a relatively common event worldwide, although its incidence varies across countries. For example, in Europe the incidence of post-term birth ranges from 0.4% in Austria to 8.1% in Denmark [1]. Post-term birth is associated with a greater incidence of peripartum complications affecting both the mother and newborn. As a result, pregnancies at risk of post-term delivery are often induced, but recommendations on the appropriate time for induction are not universal, ranging from 41 to ≥42 weeks gestation [2,3].

Little is known about the short- and long-term consequences associated with post-term birth, but two studies have reported adverse neurological outcomes in childhood [4,5]. We have recently shown in a longitudinal study of a Swedish cohort that post-term males had accelerated weight gain during childhood, and an associated increased risk of obesity in adolescence [6]. As a result, at 16 years of age the combined rate of overweight and obesity among post-term males was 47% compared to 13% for participants born at term [6].

There are however, no data on the metabolic consequences of post-term birth. Post-term fetuses are likely exposed to *in utero* stressors associated with a prolonged gestation, which may be analogous to those experienced by small-for-gestational-age (SGA) infants prior to birth and by preterm infants in the early neonatal period. Studies have shown that those born SGA or preterm are at an increased risk of adverse metabolic outcomes in adulthood, including insulin resistance, type 2 diabetes mellitus, hypertension, hyperlipidemia, stroke, as well as cancer [7,8]. As a result, this study aimed to assess whether post-term birth would affect insulin sensitivity, as well as other metabolic parameters and body composition in childhood.

## Methods

### Ethics statement

Ethics approval for this study was provided by the Northern Y Regional Ethics Committee (Ministry of Health, New Zealand) and the Auckland District Health Board Research Review Committee. Written informed consent was obtained from parents or guardians, as well as verbal or written consent from each child as was appropriate to their age.

### Participants

**Figure 1:**
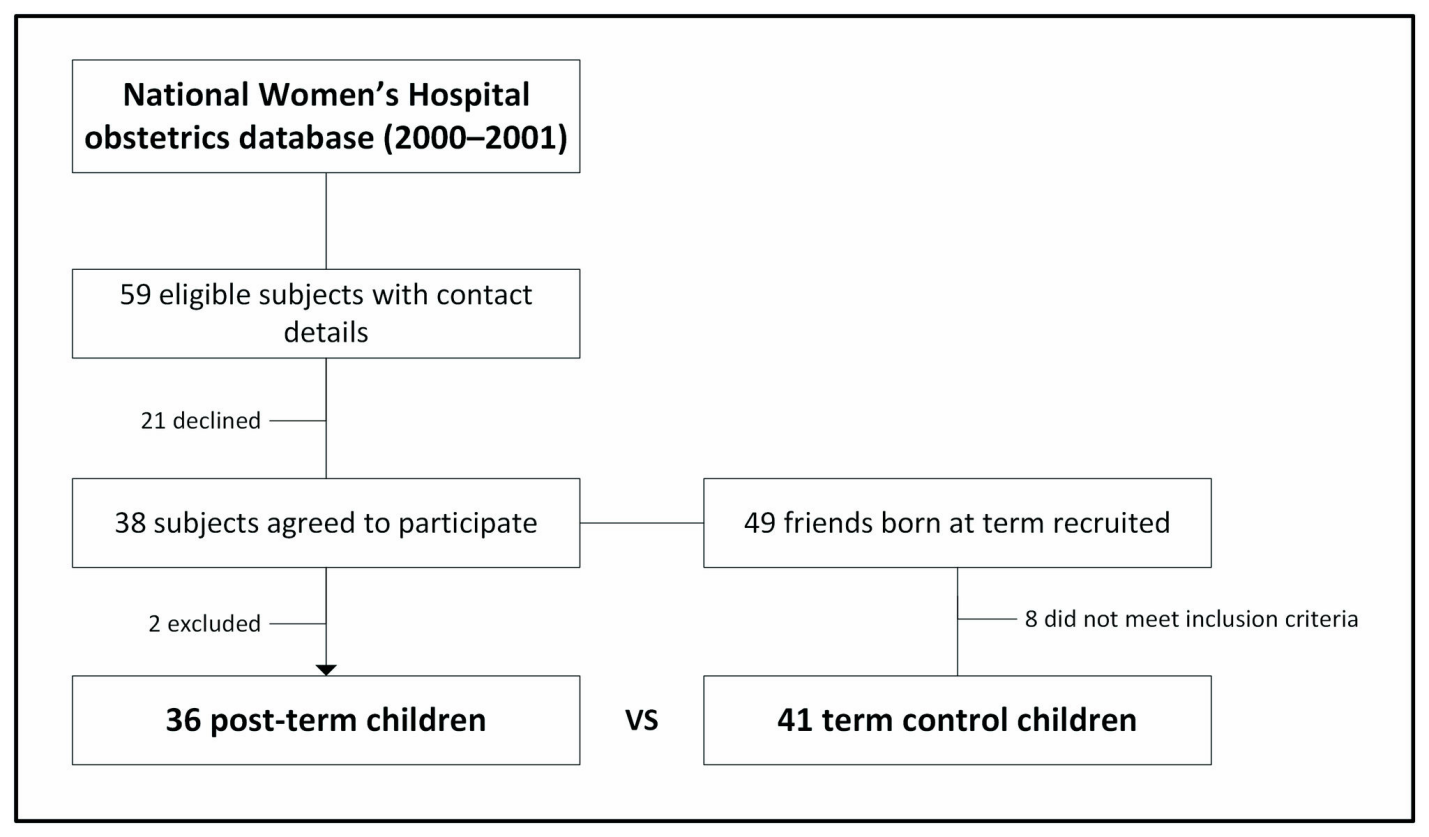
Summary of the study’s recruitment process.

Healthy, developmentally normal pre-pubertal children born from singleton pregnancies and aged 4–11 years were recruited for this study, between November 2010 and November 2011. Potential post-term participants were born at a single centre (National Women’s Health, Auckland City Hospital, Auckland, New Zealand), and identified from its obstetrics database. Control participants (term children) were friends of participants (Figure 1). Each recruited post-term child was asked to invite up to two friends born at term, so that participants in both groups were approximately matched for age and socio-economic status.

Gestational ages were determined by ultrasound scans performed <20 weeks gestation. Each scan was performed by a qualified radiologist, and all scans were subsequently re-examined and validated by the first author (AA). Data from 81 singleton pregnancies after *in vitro* fertilization (where conceptions can be accurately timed) showed that ultrasound scans in the first 20 weeks underestimated gestation length by just 2.8 days (standard error of the mean = 0.2), so that fetal age was determined to within 7 days in more than 95% of cases [9]. Nonetheless, in our study, only term children born 38–40 weeks gestation (following spontaneous labour) were recruited, to account for possible errors in gestational age estimation. This effectively created a gestational age difference of approximately two weeks separating term and post-term groups.

Exclusion criteria included signs of puberty (Tanner stage 2 breast development in girls and testicular volume >3 ml in boys or evidence of adrenarche), premature birth, being born SGA (birth weight <-2 standard deviation scores (SDS)) or as a result of *in vitro* fertilization, having genetic syndromes, receiving medication that could affect insulin sensitivity, as well as having a first-degree relative or grandparent with pre-diagnosed diabetes, the metabolic syndrome, or any of its features other than central adiposity. Children were also excluded if born to mothers with gestational diabetes, pre-eclampsia, gestational or pre-existing hypertension, chronic illnesses, or maternal drug use during pregnancy (including tobacco and alcohol).

### Clinical assessments

All children were assessed at the Maurice & Agnes Paykel Clinical Research Unit (Liggins Institute, University of Auckland). Data on each child were collected during a single visit to the clinic. A number of neonatal parameters were recorded, including birth weight, ponderal index, gestational age, and maternal age at time of delivery. Birth weight data were transformed into SDS [10].

### Primary outcome

Insulin sensitivity was assessed using a 90-minute modified frequently sampled intravenous glucose test (FSIGT), modified with insulin, and analysed using Bergman’s minimal model software [11]. Three baseline samples were drawn at -20, -10, and 0 minutes. A 25% dextrose infusion (at 0.3 g/kg) started at 0 minute and lasted for one minute. Blood samples were drawn at 2, 3, 4, 5, 6, 8, 10, 12, 14, 16, and 19 minutes. Insulin (0.015 units/kg) was then administered intravenously as a bolus at 20 minutes, and further samples were drawn at 22, 23, 24, 25, 27, 30, 35, 40, 45, 50, 60, 70, 80, and 90 minutes. No episodes of hypoglycaemia (blood glucose <4 mmol/l) were recorded in any of the participants throughout the study.

### Secondary outcomes

Children’s heights were measured using a Harpenden stadiometer. Weight and body composition data were obtained using whole-body dual-energy X-ray absorptiometry (DXA, Lunar Prodigy 2000, General Electric, Madison, WI, USA), specifically: total body fat, fat-free mass, truncal fat, and android fat to gynoid fat ratio. The latter parameters are provided by the manufacturer’s software based on an automated sectioning of specific areas of the body [12]. Studies in children have shown that proportionally greater adiposity in the upper body (i.e. android fat) is associated with an increased risk of adverse metabolic outcomes [13].

Height SDS were derived from Tanner/Whitehouse reference data [14], and weight and body mass index (BMI) SDS according to British 1990 standards [10,15]. Maternal and paternal weight and height were recorded for all participants. Mean parental BMI was calculated as the average of maternal and paternal BMI. Mid-parental height was calculated using standard formulas [16]. Ethnicity was recorded by self-report using a prioritised system, such that if multiple ethnicities were selected, the patient was assigned to a single category, following a hierarchical system of classification [17].

24-hour ambulatory blood pressure monitoring was carried out following the clinical visit, when participants were fitted with a Spacelabs 90217 monitor (Spacelabs Medical Inc., Redmond, WA, USA) on the non-dominant arm. Measurements were performed every 20 minutes from 07:00–22:00, and every 30 minutes from 22:00–07:00. Only profiles with more than 14 daytime and 7 nocturnal readings over a 24-hour period were included for analysis (as per British Hypertension Society recommendations). Parameters reported were mean arterial blood pressure, and nocturnal systolic and diastolic blood pressure dipping.

Following an overnight fast, baseline blood samples were drawn to measure serum total cholesterol, high-density lipoprotein cholesterol (HDL-C), low-density lipoprotein cholesterol (LDL-C), triglycerides, insulin-like growth factor I (IGF-I), IGF binding protein 1 (IGFBP-1), leptin, total adiponectin, androstenedione, dehydroepiandrosterone sulphate (DHEAS), glucose, insulin, highly-sensitive C-reactive protein (CRP), and uric acid concentrations. Other parameters derived from the FSIGT were the acute insulin release (insulin secretory capacity), glucose effectiveness (glucose-mediated glucose uptake), and disposition index (increase in insulin secretion to compensate for insulin resistance and maintain normoglycaemia).

### Assays

Glucose and uric acid concentrations were measured on a Hitachi 902 autoanalyser (Hitachi High Technologies Corporation, Tokyo, Japan) by enzymatic colorimetric assay (Roche, Mannheim, Germany), with an inter-assay coefficient of variation (CV) of 2.1 and 1.8%, respectively. Insulin concentrations were measured using an Abbott AxSYM system (Abbott Laboratories, Abbott Park, IL, USA) by microparticle enzyme immunoassay, with a CV of 5.7%. Total cholesterol, HDL-C, LDL-C, and triglyceride concentrations were measured using a Hitachi 902 autoanalyser, with CV of 8.9, 11.4, 10.1, and 5.3% respectively. Commercially available ELISA kits E20, E01, E07, and E09 (Mediagnost, Reutlingen, Germany) were used for quantitative determination of serum IGF-I, IGFBP-1, leptin, and adiponectin concentrations, respectively; assay sensitivities were 0.09, 0.2, 1.0, and 0.6 ng/ml, with CV of 3.1, 9.4, 6.7, and 3.0%, respectively. CRP concentrations were also measured with an ELISA kit (USCN Life Science Inc., Wuhan, China), with CV of <10%. DHEAS and androstenedione concentrations were measured using Finnigan TSQ Quantum Ultra AM triple quadruple mass spectrometer controlled by Finnigan Xcaliber software (Thermo Electron Corporation, San Jose, CA, USA); mean CV were 18.4% for DHEAS and 8.2% for androstenedione.

### Statistical analyses

Potential differences between post-term and term groups at baseline were tested using one-way ANOVA or non-parametric Kruskal-Wallis, while sex ratio and ethnic composition data were compared with Fisher’s exact tests (all in Minitab v.16, Pennsylvania State University, State College, PA, USA). Similar tests were conducted to compare post-term children included in this study to those who were not contactable or declined to participate.

Random effect mixed models were used to compare the primary and secondary outcomes between post-term and term groups. Important confounding factors were adjusted for in the analyses, including ethnicity, birth weight SDS, birth order, age, and gender. Other factors were controlled for as required, depending on the outcome response of interest: for lipids, hormones, and outcomes associated with glucose homeostasis – BMI SDS was included; for anthropometric data – the appropriate parental factor (i.e. mean parental BMI or mid-parental height); and for blood pressure parameters – height and total body fat percentage. For the primary outcome (insulin sensitivity) models were run separately for boys and girls. The interaction effect between group and gender was tested in all models, and secondary outcomes were assessed separately for boys and girls if there was indication of a differential response to post-term birth between genders.

All multivariate analyses were performed in SAS version 9.3 (SAS Institute Inc, Cary NC, USA). When necessary, response variables were log-transformed to approximate normality. All statistical tests were two-tailed and maintained at a 5% significance level. Age data are presented as means ± standard deviation. Outcome data are presented as model-adjusted means (estimated marginal means adjusted for the confounding factors in the models), with associated 95% confidence intervals.

## Results

### Participants

There were 59 post-term children born in 2000–2001 in the National Women’s Hospital obstetrics database who met the study criteria and could be traced (Figure 1). As 21 declined to participate, we recruited 38 post-term participants (Figure 1). Two participants had to be subsequently excluded: one due to maternal ill-health and another child could not be cannulated. Thus, a total of 36 post-term children (18 boys and 18 girls) were studied (Figure 1).

Post-term children who were not contactable or declined to participate were of similar birth weight (p=0.16), current age (p=0.58), and sex ratio (p=0.99) as those who participated. Through post-term children a total of 49 term controls volunteered, but 8 did not meet inclusion criteria, so that 41 participated in the study.

Children were aged 9.4 ± 1.9 years (range 4.0–11.9 years); age, birth weight, ponderal index, sex ratio, and ethnic composition were all similar between post-term and term groups (Table 1). In addition, there were no differences in parental variables between groups, namely maternal age, pre-pregnancy maternal BMI, and mean parental BMI at the time of the study (Table 1).

**Table 1:** Baseline characteristics of children born post-term or at term.

		**Post-term children**	**Term children**	**p-value**
**n**		36	41	
**Maternal age at childbirth** (years)		32.5 ± 5.2	33.6 ± 4.7	0.39
**Maternal pre-pregnancy BMI** (kg/m^2^)		25.3 ± 6.0	24.7 ± 5.2	0.62
**Mean parental BMI** (kg/m^2^)		28.1 ± 4.4	27.8 ± 4.9	0.65
**Age** (years)		9.7 ± 1.3	9.1 ± 2.2	0.33
**Gestational age** (weeks)		42.2 ± 0.2	39.5 ± 0.7	**<0.0001**
**Birth weight SDS**		0.53 ± 0.95	0.35 ± 0.83	0.39
**Ponderal index**		25.8 ± 2.8	27.0 ± 4.4	0.34
**Sex ratio** (boys)		50%	66%	0.17
**Ethnicity**	New Zealand European	72%	83%	0.28
	Maori / Pacific Islander	22%	10%	
	Indian	6%	7%	

Data are means and standard deviations.

### Insulin sensitivity and other parameters of glucose homeostasis

**Figure 2:**
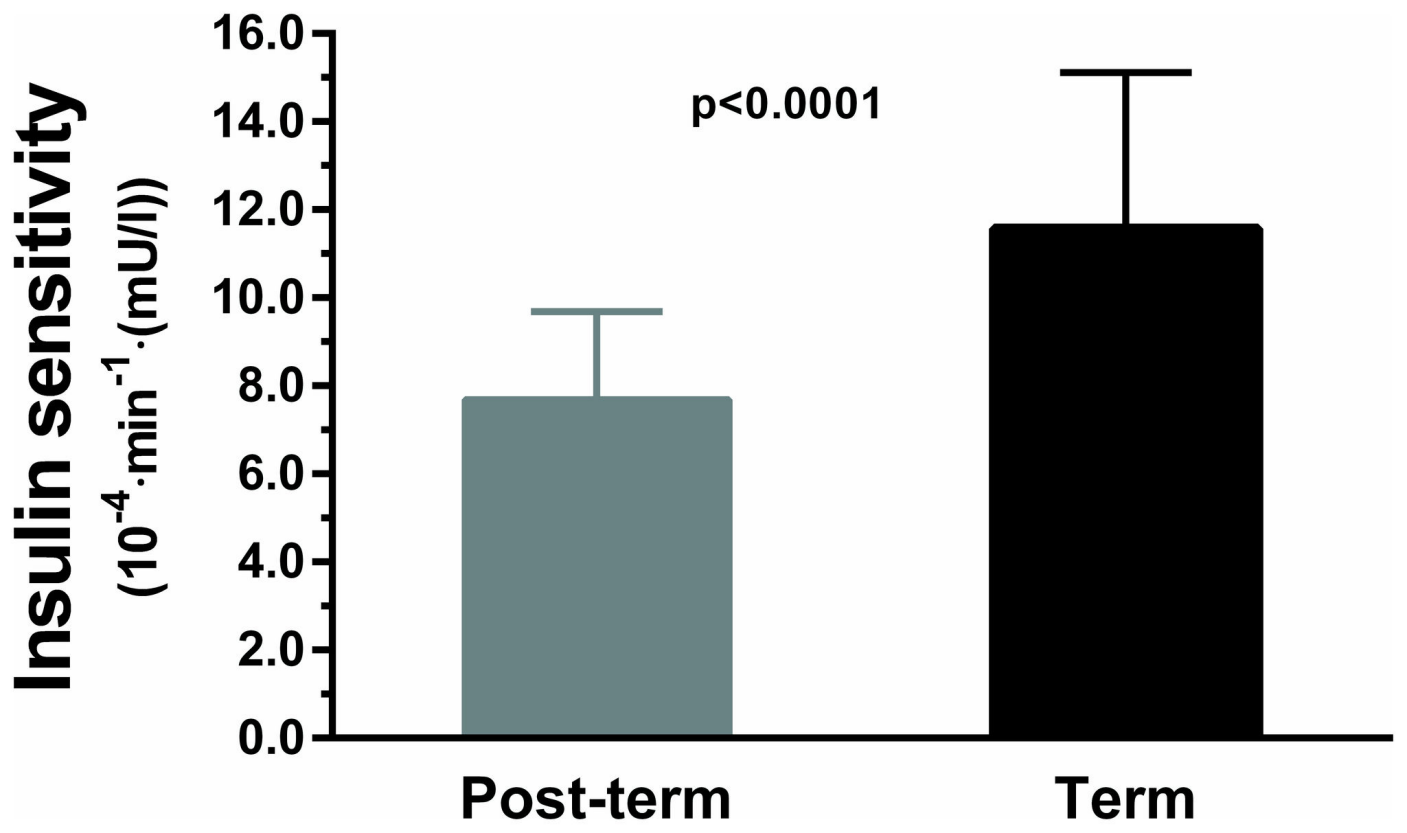
Insulin sensitivity (primary outcome) among children born post-term or at term. Data are means and 95% confidence intervals adjusted for other confounding factors in the multivariate models.

Insulin sensitivity was 34% lower in post-term children compared to term children (7.7 vs. 11.6 x10^-4^·min ^-^1·(mU/l)) (Figure 2). Glucose effectiveness was also 28% lower among post-term children in comparison to those born at term (Table 2). However, the lower insulin sensitivity was compensated by an acute insulin response that was 38% higher among post-term children (Table 2). Disposition index, fasting glucose and insulin concentrations were not different in post-term and term children (Table 2).

**Table 2:** Secondary outcomes among children born post-term or at term.

		**Post-term children**	**Term children**	**p-value**
**n**		36	41	
**Glucose homeostasis**	Acute insulin response (mU/l)	418 (339–516)	304 (235–392)	**0.037**
	Glucose effectiveness (10^-2^/min)	2.25 (1.52–3.33)	3.11 (2.01–4.83)	**0.047**
	Disposition index	3539 (2406–5205)	3576 (2271–5630)	0.95
	Fasting insulin (mU/l)	5.72 (4.38–7.49)	5.20 (3.76–7.19)	0.43
	Fasting glucose (mg/dl)	86.1 (82.9–89.2)	87.2 (83.4–91.0)	0.43
**Hormone concentrations**	Leptin (ng/ml)	6.96 (4.99–8.92)	4.49 (2.13–6.86)	**0.008**
	Adiponectin (μg/ml)	7.82 (4.64–9.20)	10.75 (9.32–12.17)	**0.005**
	IGF-I (ng/ml)	204 (168–240)	200 (157–243)	0.78
	IGFBP-1 (ng/ml)	10.6 (7.8–13.5)	17.6 (14.6–20.5)	**0.002**
	Androstenedione (nmol/l)	0.44 (0.33–0.54)	0.59 (0.48–0.70)	0.055
	DHEAS (nmol/l)	4.59 (2.53–6.66)	6.12 (3.59–8.64)	0.11
**24-hour blood pressure monitoring**	Mean arterial pressure (mmHg)	73.4 (70.0–76.8)	76.2 (72.0–80.5)	0.09
	Nocturnal systolic dipping (%)	9.7 (6.2–13.2)	13.5 (9.2–17.8)	**0.027**
	Nocturnal diastolic dipping (%)	15.6 (11.1–20.1)	19.4 (13.8–25.1)	0.079
**Inflammatory markers**	CRP (ng/ml)	457 (63–851)	490 (6–974)	0.86
	Uric acid (μmol/l)	229 (211–247)	201 (181–220)	**0.033**

Data are means and 95% confidence intervals adjusted for other confounding factors in the multivariate models.

Insulin sensitivity was 33% lower among post-term boys (8.5 vs 12.7 x10^-4^·min^-1^(mU/l); p=0.001) and 44% lower among post-term girls (5.6 vs 10.0 x10^-4^·min^-1^(mU/l); p=0.005). However, a significant compensatory increase in acute insulin response was only observed in post-term boys compared to term counterparts (531 vs 347 mU/l; p=0.039), but not among post-term girls (367 vs 304 mU/l; p=0.49).

### Anthropometry

**Figure 3:**
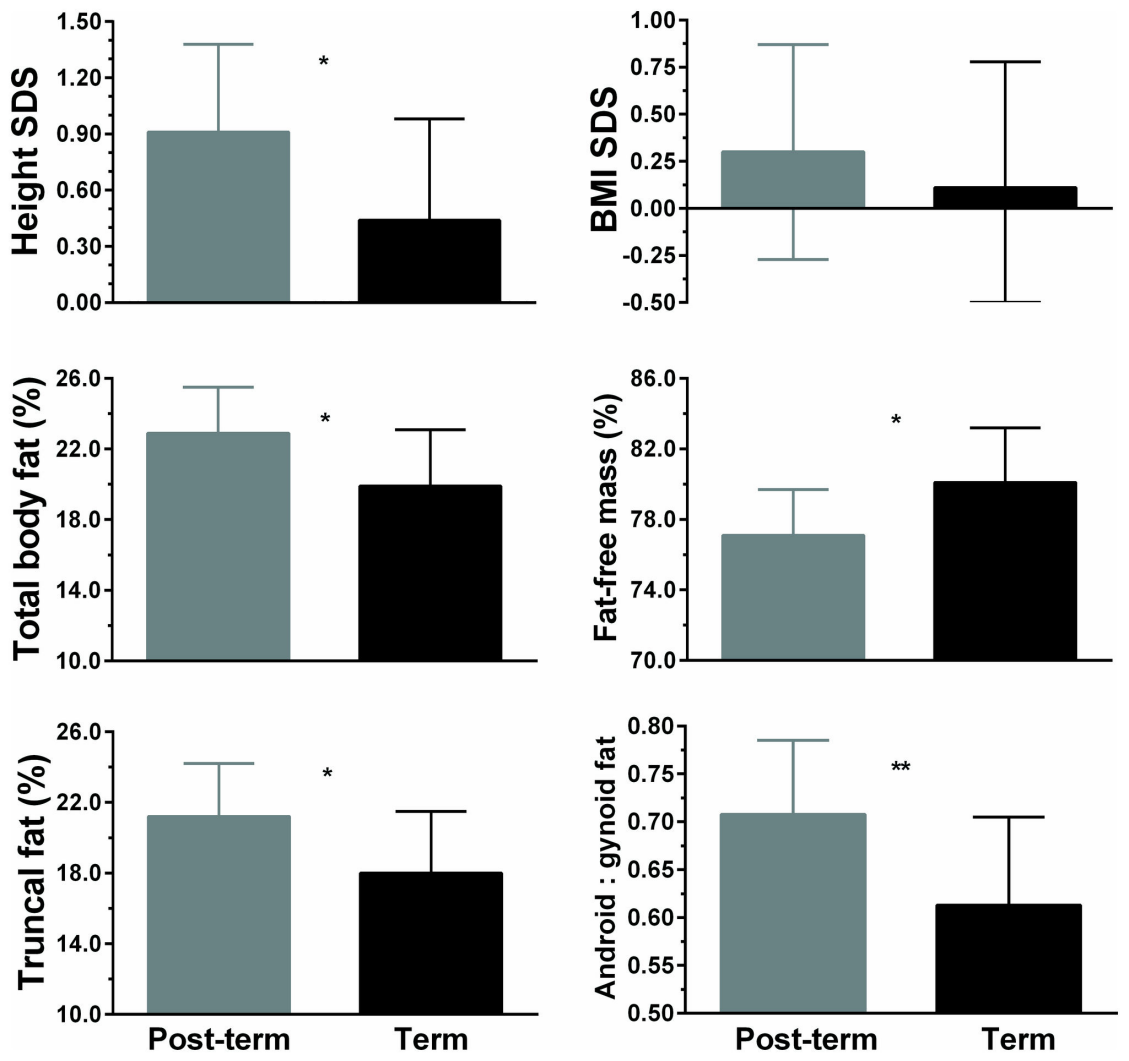
Anthropometric parameters among children born post-term or at term. Data are means and 95% confidence intervals adjusted for other confounding factors in the multivariate models. *p<0.05 and **p<0.01 for post-term vs term children.

Although post-term and term children were of similar BMI SDS (0.30 vs 0.11; p=0.45), there were considerable differences in adiposity and fat distribution between the two groups (Figure 3). Post-term children had greater body fat (22.9 vs 19.9%; p=0.014) and lower fat-free mass (77.1 vs 80.1%; p=0.014), as well as more truncal fat (21.2 vs 18.0%; p=0.017) and greater android to gynoid fat ratio (0.71 vs 0.61; p=0.007) (Figure 3). In addition, children born post-term were approximately 0.5 SDS taller (p=0.022; Figure 3). However, this pattern was mostly driven by their increased adiposity, so that addition of body fat into the statistical model removed the significant difference in height between the two groups (p=0.20).

### Blood pressure

The 24-hour ambulatory blood pressure monitoring showed that post-term children experienced lower nocturnal blood pressure dipping. This occurred for systolic blood pressure dipping, with a similar trend also observed for diastolic dipping (Table 2).

### Hormonal profile & inflammatory markers

Post-term children had higher leptin (+55%) and lower adiponectin (-37%) concentrations, as well as lower serum IGFBP-1 concentrations (-40%; Table 2). DHEAS concentrations were not different among groups (p=0.11), but post-term children tended to have lower androstenedione concentrations (-25%; Table 2). Among inflammatory markers, post-term children had higher serum uric acid concentrations (+14%; Table 2).

### Lipid profile

Overall, children born post-term had a less favourable fasting lipid profile compared to term children (Table 3). However, there was a differential response between genders, so that the less favourable lipid profile was observed only among post-term boys but not girls (Table 3). Specifically, post-term boys had higher total cholesterol (+29%) and LDL-C (+53%) concentrations, tended to have higher triglyceride concentrations (+40%), and also had higher total cholesterol to HDL-C ratio (+30%) compared to term controls (Table 3).

**Table 3:** Lipid profiles among children born post-term or at term.

		**Post-term children**	**Term children**	**p-value**
**Whole cohort**	n	36	41	
	Total cholesterol (mmol/l)	3.84 (3.27–4.40)	3.29 (2.62–3.96)	**0.034**
	LDL-C (mmol/l)	2.13 (1.72–2.55)	1.67 (1.17–2.17)	**0.016**
	Triglycerides (mmol/l)	0.81 (0.64–0.98)	0.69 (0.49–0.89)	0.12
	HDL-C (mmol/l)	1.27 (1.04–1.50)	1.23 (0.95–1.50)	0.69
	Total cholesterol : HDL-C	3.19 (2.73–3.66)	2.68 (2.11–3.25)	**0.037**
**Boys**	n	18	27	
	Total cholesterol (mmol/l)	4.04 (3.73–4.71)	3.14 (2.32–3.97)	**0.018**
	LDL-C (mmol/l)	2.23 (1.74–2.71)	1.46 (0.85–2.06)	**0.006**
	Triglycerides (mmol/l)	0.84 (0.67–1.05)	0.60 (0.47–0.76)	0.055
	HDL-C (mmol/l)	1.32 (1.03–1.60)	1.24 (0.89–1.59)	0.61
	Total cholesterol : HDL-C	3.32 (2.73–3.90)	2.56 (1.83–3.30)	**0.048**
**Girls**	n	18	14	
	Total cholesterol (mmol/l)	3.57 (2.80–4.34)	3.88 (3.04–4.71)	0.49
	LDL-C (mmol/l)	2.01 (1.52–2.49)	2.22 (1.70–2.75)	0.46
	Triglycerides (mmol/l)	0.78 (0.53–1.03)	0.71 (0.44–0.97)	0.60
	HDL-C (mmol/l)	1.17 (0.92–1.42)	1.30 (1.03–1.57)	0.36
	Total cholesterol : HDL-C	3.21 (2.65–3.77)	2.85 (2.21–3.49)	0.32

Data are means and 95% confidence intervals adjusted for other confounding factors in the multivariate models.

### New Zealand Europeans

As New Zealand Europeans made up approximately three-quarters of participants, a subgroup analyses could be carried out with the exclusion of other ethnicities. These data are provided in a supplementary table (Table S1). Overall trends remained, but statistical power was considerably reduced with the reduction in *n* (Table S1). Still, insulin sensitivity was 30% lower in post-term than in term groups (8.7 vs 12.5 x10^-4^·min ^-^1·(mU/l); p=0.003) (Table S1). In addition, compared to term controls, post-term children of New Zealand European ethnicity had lower systolic blood pressure dipping, higher leptin concentrations, lower IGFBP-1 concentrations, and tended to have greater abdominal adiposity and higher uric acid concentrations (Table S1).

## Discussion

This study shows that pre-pubertal children born post-term have lower insulin sensitivity in comparison to term children. Further, post-term children also displayed other early markers of the metabolic syndrome such as greater body fat and central adiposity, lower nocturnal blood pressure dipping, higher serum uric acid concentrations, as well as a less favourable lipid profile among post-term boys.

The metabolic syndrome comprises a combination of insulin resistance with or without glucose intolerance with any two of the following: central obesity, high blood pressure, dyslipidemia, pro-inflammatory state, or prothrombotic state [18]. A reduction in insulin sensitivity alone can predict the metabolic syndrome in adulthood [19], which in turn is an independent risk factor for later mortality from coronary heart disease and cerebrovascular disease [20]. The observed reduction in insulin sensitivity was similar to that observed among pre-pubertal children born SGA or preterm [21,22]. SGA children have insulin resistance that starts in childhood [21] and persists through early adulthood [23], being associated with an increased risk for various metabolic disorders later in life [7]. Infants born preterm also demonstrate a similar reduction in insulin sensitivity in childhood [22], which we have recently shown to persist into mid-adulthood [8]. Importantly, the lower insulin sensitivity in post-term children was not due to adrenarche, as indicated by higher adrenal androgen levels among term children. Thus, the reduction in insulin sensitivity we observed in children born post-term may persist into adult life.

Whole body glucose uptake is dependent upon three factors: insulin secretion (acute insulin response), insulin action (insulin sensitivity), and glucose uptake independent of insulin (glucose effectiveness) [24]. Severe defects in at least of two of these factors are necessary for the development of type 2 diabetes mellitus [24]. We observed impairments in two factors (insulin sensitivity and glucose effectiveness), indicating that post-term children may be at increased risk of later type 2 diabetes mellitus.

We also observed an overall increase in insulin secretion, particularly among post-term boys. A similar trend was observed among girls, but the increase in acute insulin response was of lesser magnitude and not significant likely due to reduced statistical power (lower *n* for control girls). With the greater insulin secretion, there was an expected reduction in serum IGFBP-1 concentrations amongst post-term children. Insulin resistance leads to a compensatory increase in portal insulin secretion, which suppresses IGFBP-1 concentrations [25]. Hyperinsulinemia and low IGFBP-1 are also associated with increased likelihood of developing cardiovascular disease [26]. The less favourable lipid profile observed amongst post-term boys was still within the normal range, so that the importance of these findings is unknown. However, blood lipids levels in childhood track into adulthood [27], and could precede later dyslipidemia. Furthermore, post-term children had higher concentrations of uric acid, which is also a marker of cardiovascular disease, metabolic syndrome, and type 2 diabetes mellitus [28]. A follow-up study showed that elevated uric acid levels in childhood are predictive of higher blood pressure in adult life [29].

Further, post-term children had increased body fat, lower fat-free mass, and greater central adiposity than term controls. The elevated abdominal adiposity in particular, is associated with cardiovascular disease in adulthood [30]. There was also an adverse adipokine profile in post-term children, characterized by higher leptin and lower adiponectin concentrations, consistent with a greater central fat distribution and insulin resistance [31]. These proteins are secreted by adipose tissue [32], with high leptin concentrations (leptin resistance) and low adiponectin being both associated with the metabolic syndrome [32,33]. Interestingly, we recently studied a Swedish cohort longitudinally from birth to 16 years of age, and post-term males displayed greater adiposity that markedly increased in adolescence [6]. Nearly half of post-term adolescent males were overweight or obese, and were on average 11 kg heavier than those born at term [6]. Interestingly, both sexes were adversely affected by post-term birth in this current study. In light of our earlier study in adolescents born post-term, it is conceivable that post-term males may experience more marked changes in body composition and metabolism over time. Nonetheless, our data suggest that insulin resistance in post-term children precedes the major changes in body composition observed in adolescence [6].

Post-term children also had markedly lower nocturnal systolic blood pressure dipping, with a similar trend observed for diastolic dipping. This observation is important, as the normal reduction in nocturnal blood pressure dipping is a risk factor for cardiovascular mortality in adults, irrespective of overall blood pressure [34]. There is a normal reduction in nocturnal blood pressure in children and adults that is >10% [35], but post-term children already displayed a mean systolic dipping below this threshold. Unfortunately, there are no long-term data on the effects of reduced nocturnal dipping in childhood, which would better clarify the long-term cardiovascular consequences to our post-term cohort.

The underlying factors associated with the observed metabolic changes in post-term children are unclear. We speculate that the factors underpinning the metabolic programming in children born post-term may be associated with genetic inheritance or an adverse fetal environment in late gestation, which is unlikely to be associated with nutritional compromise. The Dutch Famine study showed an increased risk of glucose intolerance in adulthood among those exposed to famine in late gestation compared to early gestation [36]. However, an under-nutrition insult *in utero* is unlikely to occur in most prolonged pregnancies, as our post-term infants had comparable birth weights (when adjusted for gestational age) and ponderal indices to term children.

Whilst the cause of the adverse environment is unclear, there are changes in placental histology seen in post-term pregnancies [37]. Nonetheless, post-term birth is common in first-degree relatives, suggesting an element of genetic inheritance [38]. Therefore, a common genetic variant or epigenetic modification may possibly lead to prolonged gestation, and, at least in part, underpin the observed metabolic changes in post-term children. In addition, prolonged gestation could also be associated with an increased stress response in the fetus (e.g. higher glucocorticoid levels) [39], which may lead to the metabolic changes we observed later in post-natal life. However, animal models of excessive glucocorticoid exposure are usually associated with reduced birth weight and alterations in the hypothalamic-pituitary-adrenal axis [39], neither of which was seen in our cohort.

In conclusion, our study shows that pre-pubertal children born post-term have lower insulin sensitivity and a number of early markers of the metabolic syndrome. These include increased body fat, greater central adiposity, lower nocturnal blood pressure dipping, and higher uric acid concentrations, as well as a less favourable lipid profile in post-term boys. Interestingly, the range of adverse metabolic outcomes in post-term children greatly exceeds those observed in SGA or preterm children. Thus, our findings in post-term children could have major implications for the management of prolonged pregnancies. It is important to study post-term cohorts in adulthood to adequately identify the long-term health risks associated with prolonged gestation. Further, future studies are necessary to establish the underlying causes and mechanisms of these changes in post-term children.

## Supporting Information

Table S1 Study outcomes among children of New Zealand European ethnicity who were born post-term or at term. Data are means and 95% confidence intervals adjusted for other confounding factors in the multivariate models.
(PDF)
