# Supplementary material for: Pre-Pubertal Children Born Post-Term Have Reduced Insulin Sensitivity and Other Markers of the Metabolic Syndrome
Source: PLoS One. 2013 Jul 1;8(7):e67966. doi: 10.1371/journal.pone.0067966 (PMC3698136; doi:10.1371/journal.pone.0067966)
Supplement: Table S1 — Data are means and 95% confidence intervals adjusted for other confounding factors in the multivariate models. (PDF) [file pone.0067966.s001.pdf]

Table S1. Study outcomes among children of New Zealand European ethnicity who were born post-term or at term. Data are means and 95% confidence intervals adjusted for other confounding factors in the multivariate models.

	Post-term children	Term children	P-value
**n**	26	34	
**Anthropometry**			
Height SDS	0.66 (0.34–0.98)	0.37 (0.07–0.66)	0.20
BM ISDS	0.33 (-0.06–0.71)	0.25 (-0.13–0.64)	0.79
Total body fat (%)	23.2 (21.3–25.0)	21.1 (19.3–23.0)	0.14
Fat-free mass (%)	76.8 (75.0–78.7)	78.9 (77.0–80.7)	0.13
Truncal fat (%)	20.5 (18.4–22.6)	18.7 (16.7–20.8)	0.24
Android fat to gynoid fat ratio	0.71 (0.66–0.77)	0.64 (0.59–0.69)	*0.056*
**Glucose homeostasis**			
Insulin sensitivity (x10^{-4}·min^{-1}·(mU/l))	8.7 (7.4–10.3)	12.5 (10.8–14.4)	**0.003**
Acute insulin response (mU/l)	269 (211–342)	217 (172–272)	0.22
Glucose effectiveness (10^{-2}/min)	1.69 (1.26–2.27)	2.41 (1.86–3.11)	*0.088*
Disposition index	2403 (1802–3206)	2393 (1844–3106)	0.98
Fasting insulin (mU/l)	4.51 (3.67–5.53)	4.09 (3.36–4.99)	0.51
Fasting glucose (mg/dl)	82.8 (80.6–85.1)	84.7 (82.5–86.8)	0.27
**Hormone concentrations**			
Leptin (ng/ml)	5.95 (4.81–7.08)	4.19 (3.16–5.22)	**0.032**
Adiponectin (µg/ml)	8.13 (6.83–9.67)	9.88 (8.38–11.6)	0.12
IGF-I (ng/ml)	191 (165–217)	189 (164–214)	0.92
IGFBP-1 (ng/ml)	9.92 (7.79–12.6)	15.4 (12.3–19.4)	**0.013**
Androstenedione (nmol/l)	0.34 (0.27–0.44)	0.50 (0.40–0.62)	**0.034**
DHEAS (nmol/l)	2.26 (1.46–3.49)	3.96 (2.69–5.83)	*0.074*
**24-hour blood pressure monitoring**			
Mean arterial pressure (mmHg)	75.4 (72.9–78.0)	78.5 (75.9–81.0)	0.11
Nocturnal systolic dipping (%)	7.5 (5.1–10.0)	11.3 (8.9–13.8)	**0.041**
Nocturnal diastolic dipping (%)	14.4 (11.4–17.5)	17.1 (14.0–20.2)	0.24
**Inflammatory markers**			
CRP (ng/ml)	228 (81–374)	240 (95–385)	0.91
Uric acid (µmol/l)	224 (202–245)	195 (174–216)	*0.061*
**Lipid profile**			
Total cholesterol (mmol/l)	4.26 (3.81–4.71)	3.95 (3.56–4.35)	0.33
LDL-C (mmol/l)	2.52 (2.20–2.85)	2.27 (1.98–2.56)	0.27
Triglycerides (mmol/l)	0.78 (0.66–0.91)	0.74 (0.62–0.85)	0.61
HDL-C (mmol/l)	1.31 (1.14–1.48)	1.30 (1.14–1.45)	0.92
Total cholesterol : HDL-C	3.36 (3.03–3.68)	3.11 (2.82–3.41)	0.29
